# Discursive strategies of Chinese elders in intergenerational conflicts: A critical discourse analysis of mediation television programs in China

**DOI:** 10.1371/journal.pone.0320909

**Published:** 2025-06-04

**Authors:** Chen Cai, Heng Hu

**Affiliations:** School of Jiayang, Zhejiang Shuren University, Hangzhou, Zhejiang, China; The University of Edinburgh, UNITED KINGDOM OF GREAT BRITAIN AND NORTHERN IRELAND

## Abstract

Research on conflict discourse highlights the value of a cross-cultural perspective and the need to explore its features across different cultural contexts. However, there is limited research on conflict discourse among Chinese elders, particularly within the framework of building an Age-Friendly Society. To address this gap, this study adopts Fairclough’s dialectical-relational approach in critical discourse analysis to examine the strategies Chinese elders employ in intergenerational conflicts. Drawing on Li’s framework of conflict strategies—competition, cooperation, avoidance, and compromise—this study identifies how Chinese elders use discourse to safeguard their rights and foster mutual understanding within family interactions. The findings reveal that these four strategies serve as discursive means for elders to achieve self-fulfillment, protect their rights, and sustain family harmony amidst social change. This study offers a systematic analysis of conflict resolution strategies in intergenerational discourse, highlighting the nuanced equilibrium that elders navigate between upholding traditional values and accommodating the evolving dynamics of contemporary family life.

## Introduction

Amidst the global phenomenon of population aging, the notion of an Age-Friendly Society (AFS) has emerged as a cornerstone of contemporary social policy, underscoring the necessity of cultivating environments that cater to the multifaceted needs of the elderly [1]. Originating from the World Health Organization’s framework, AFS places a strong emphasis on fostering inclusive communities that not only safeguard the health and dignity of older adults but also actively encourage their social participation [2]. Within this broader context, China, confronted with the pressing realities of a rapidly aging population, has strategically integrated the principles of AFS into its national agenda as a means of addressing the complex challenges associated with demographic shifts [3]. Consequently, scholarly inquiry has expanded beyond macro-level policy analysis to examine the intricate dynamics of family life, wherein the vast majority of older adults experience their later years [4,5,6]. As the fundamental social unit, the family not only provides the primary context for aging experiences but also serves as a dynamic site where intergenerational relationships, caregiving responsibilities, and cultural expectations intersect, rendering it an indispensable domain for academic investigation.

To enhance the quality of life for Chinese elders, family conflicts involving elderly members have become an important yet under-researched topic. Family conflict, often linked to generational differences and changing societal roles, can significantly affect the mental and physical wellbeing of everyone involved [7]. Current studies have highlighted the causes and signs of these conflicts, showing the need for effective intervention strategies [8,9]. However, one crucial aspect that remains insufficiently explored is the role of language in these conflicts. Language is not just a way to communicate; it shapes perceptions, asserts identities, and negotiates power in family relationships [10]. In the Chinese context, where Confucian ideals of filial piety exist alongside modern changes in family structures, understanding how elderly individuals use language to negotiate their needs and rights in conflicts holds unique cultural importance [11]. Therefore, given this complexity, critical discourse analysis (CDA) becomes particularly relevant as it examines not only the forms and structures of language but also the connections between language, power, and ideology [12]. This perspective allows us to uncover the socio-cultural factors underlying family conflicts and their impact on the elderly.

Above all, this study conducts a CDA, specifically Fairclough’s dialectical-relational approach to analyze the language used by Chinese elders in family conflicts. The Fairclough’s approach to CDA is chosen because it offers a systematic framework for examining how language constructs, maintains, and challenges power relations within family relationships. Unlike other approaches, Fairclough’s CDA emphasizes the dialectical relationship between language and social structures, allowing us to explore how language not only reflects but also shapes social realities, particularly in contexts where power imbalances exist within families [12]. This approach is especially relevant as it helps uncover how discourse reinforces power dynamics. In addition, the study can help understand how they assert their rights, negotiate roles, and handle conflicts in a society that is rapidly changing by investigating the ways Chinese elders use language. Therefore, Fairclough’s dialectical-relational approach shows how cultural norms, history, and social factors influence communication. Through this analysis, the study aims to offer a deeper understanding of the balance between tradition, modern values, and personal choice in China’s changing family dynamics.

## Literature review

Conflict discourse refers to a state of opposition, negation, or debate that arises between the speaker and the listener due to differences in opinions, perspectives, or stances [13]. Xu and Li [14] suggest that conflict discourse originates from contrasting attitudes and emotions and is commonly observed in verbal behaviors and events such as debates, disagreements, rebuttals, and objections. Historically, conflict discourse has been marginalized as a form of speech behavior, receiving insufficient attention due to academic bias toward impoliteness [15]. Research in conflict discourse began with marital disputes and later expanded to groups such as children and adolescents [16,17]. With the rise of sociolinguistics, research on conflict discourse has further extended to institutional, media, and online environments [18,19].

Research on elder conflict discourse examines how older adults communicate during family disagreements, focusing on the characteristics of conflict speech acts, discursive strategies, pragmatic effects, and the identities of communicative participants [20]. A central focus in the field has been the discursive strategies that older adults use in family interpersonal conflicts, as these strategies directly relate to their communication behaviors, psychological states within family relationships, and how they manage and express conflict through language [21]. Qiao [22] found that older adults often adopt mitigating language strategies, such as avoiding direct confrontation, using vague expressions, and employing euphemisms to maintain family harmony. However, other research suggests that in some situations, older adults may use more direct and assertive language strategies to defend their rights [23]. These varied strategies reflect changes in the social status of older adults within the family and their awareness of their rights. Additionally, some studies highlight the influence of cultural factors on elders’ language strategies. For example, older adults in rural China may prefer traditional family values to mediate conflicts [24]. These studies suggest that elders employ diverse discursive strategies in family conflicts, which not only reflect their perceived status and rights within the family but are also shaped by cultural context. The above studies provide an important foundation for understanding the communicative behaviors of older adults in family relationships.

However, significant research gaps remain, especially in the context of China, where the characteristics and motivations of older adults in family conflicts require further exploration. In recent years, China’s rapid development has widened the generational gap in thinking, values, and behaviors between older and younger generations, leading to intergenerational conflict [25]. With China’s aging population, conflicts between elderly parents and adult children have become a major family issue. According to Zhai and Feng [26], although family remains the primary source of emotional support and care for Chinese elders, their influence within the family has significantly declined. This area of research is crucial for understanding how Chinese elders employ discursive strategies to safeguard their rights and foster mutual understanding among family members.

This study aims to analyze the strategic characteristics of Chinese elders in family conflicts from the perspective of CDA and to explore the power dynamics underlying these strategies, addressing the above research gaps. Additionally, it will examine the ideological foundations of these strategies, particularly how they reflect, uphold, or challenge existing social power relations and perceptions of aging. The aim of the current study is expected to enhance our understanding of conflict resolution mechanisms and inform policy interventions that better align with the communicative realities and needs of older adults. The research questions are as follows:

1. What discursive strategies do Chinese elders use in family conflicts?
2. How do elders negotiate their power relations in family interpersonal conflicts through discursive strategies?

## Theoretical framework

CDA, as described by Fairclough [27, p. 12], studies ‘social identities, social relations, knowledge, and belief,’ aiming to explain social issues by illustrating the effects of underlying structures and mechanisms rather than just describing them. CDA investigates how discourse reinforces existing power structures and sustains social hierarchies that lead to persistent dominance and marginalization [28]. In other words, CDA combines critique with explanation to demonstrate how discourse contributes to shaping social reality [29]. Therefore, as a transdisciplinary field, CDA is dedicated to addressing social problems, often starting with semiotic data analysis.

Applying CDA to analyze intergenerational conflict discourses involving elders is crucial for several reasons. CDA enables a deeper understanding of how power dynamics and social ideologies shape communication between generations, revealing how these dynamics are negotiated through language and discourse. Elders often face challenges to their traditional authority from younger generations, especially in rapidly changing societies. Additionally, linking CDA to discursive strategies and using these strategies as an analytical tool enables a more nuanced analysis of the linguistic features employed in conflicts. Through an examination of discourse, researchers can identify the impact of societal changes, such as economic reforms and modernization, on elders' roles and perceptions within the family.

Currently, research on elder conflict discourse from the perspective of CDA has gained significant attention and is widely recognized in academic studies [8,30]. This growing body of research focuses on how language reflects and shapes conflicts among older adults, particularly in relation to power dynamics, social roles, and family relationships. Researchers are increasingly focusing on CDA to gain a deeper understanding of the underlying power dynamics and social impacts in conflicts because CDA systematically explores how language functions as a tool for constructing, reinforcing, or challenging power relations within social interactions [30]. Through the analysis of discourse, CDA unveils hidden power structures, ideologies, and the mechanisms through which language shapes social realities, establishing itself as an effective approach for uncovering the complexities inherent in conflict situations. The increasing significance of CDA in contemporary study highlights its role in examining how power structures, social norms, and ideologies shape discourse. Fairclough’s CDA framework provides a comprehensive approach to analyzing discourse at both macro and micro levels. At the macro level, CDA explores the broader socio-cultural and ideological contexts that influence discourse production and interpretation. At the micro level, it examines specific linguistic choices and discursive strategies that reflect and reinforce power relations.

Central to Fairclough’s approach is the theory of power, which conceptualizes power as both relational and discursive. Power is not only exercised through overt dominance but also through subtle, everyday language practices that shape social identities, relationships, and systems of knowledge. Fairclough distinguishes between power in discourse and power behind discourse. Power in discourse refers to how power operates within interactions, influencing who speaks, how they speak, and whose voices are heard or marginalized. Power behind discourse examines the social structures and institutions that shape discourse practices, embedding power relations within broader societal frameworks.

In addition to the theory of power, Fairclough’s dialectical-relational approach to CDA is important in understanding the relationship between discourse and social structures. This approach highlights that discourse is both a product of and a contributor to social reality, operating within a dialectical relationship where discourse shapes and is shaped by social practices, structures, and relations. Fairclough identifies three key dimensions of this approach: text (the linguistic features and organization of discourse), discourse practice (the processes of production, distribution, and consumption of texts), and sociocultural practice (the broader social and cultural structures that influence discourse). This three-dimensional model allows researchers to analyze discourse at multiple levels, examining how linguistic choices reflect and reproduce social inequalities and power relations.

Applying Fairclough’s approach to this study, we first identify how discourse surrounding older adults is shaped by societal expectations and power dynamics. This study contributes to the theoretical understanding of discourse and aging through an examination of how language reflects and reinforces age-related power structures. For instance, Dubus [31] found that older adults’ awareness of their identity influences their engagement in power negotiations. Similarly, Fingerman et al. [8] observed that intergenerational value differences create tension, contributing to social conflicts. These findings align with Fairclough’s notion that discourse is both socially shaped and socially constitutive. Furthermore, this study extends Fairclough’s framework through the integration of cultural and social dimensions into discourse analysis. As Pham et al. [32] demonstrated, cultural norms play a crucial role in shaping conflict resolution and guiding strategic choices. The analysis highlights the intersection of aging, power, and cultural variation, offering new insights into the broader implications of discourse on social relations.

This study examines how older adults navigate discourse, negotiate power, and select conflict resolution strategies in response to social and ideological influences by integrating Fairclough’s CDA framework. This approach enables a deeper understanding of how discourse constructs and reflects broader societal structures and power relations.

## Research methods

### Discursive strategies and critical discourse analysis

The discursive strategies employed in this study are based on [33], offering a structured framework for analysis. CDA reveals how language reflects and reinforces social hierarchies and inequalities. It highlights how elders use discursive strategies to maintain their social status and resist marginalization, thus contributing to a broader understanding of social justice and equity. The strategies identified by [33] provide clear definitions and examples by incorporating CDA, thereby strengthening the analysis of elders’ involvement in power dynamics. Li’s framework is particularly suitable for this project as it systematically categorizes strategies that reflect both explicit and implicit dimensions of discourse, allowing for a nuanced examination of power dynamics and social interactions in this specific context. This study explores four primary conflict discursive strategies, each reflecting different approaches to negotiating power and maintaining social relationships.

Competition is a highly assertive and non-cooperative conflict management strategy. In interpersonal conflicts, a competitive strategy often serves as a form of self-protection, where one party asserts their position strongly to achieve their desired outcome without considering the interests of the other party.

Cooperative strategies, on the other hand, are communication approaches that aim to build consensus, foster understanding, and seek common ground. This strategy emphasizes non-confrontational language and behavior, aiming to avoid blame and negative judgments. Cooperative strategies diminish tension and hostility, thereby establishing conditions that foster further communication and enhance the potential for effective problem-solving.

The compromise strategy seeks to resolve conflict by finding a middle ground through mutual concessions. It is often regarded as a semi-thorough solution, where both parties make concessions to reach an agreement. People who adopt this strategy maintain a moderate focus on both their own interests and those of others, reducing disagreements by identifying solutions that both sides can accept.

Lastly, the avoidance strategy refers to a conflict management approach that is neither cooperative nor competitive, often failing to satisfy the interests of either party. Individuals using this strategy tend to ignore disagreements and conflicts, distancing themselves from the situation and allowing the conflict to play out on its own.

When analyzed through CDA, these four strategies provide a comprehensive framework for understanding how language and discourse influence interpersonal conflicts. This approach enriches the analysis by revealing the nuanced ways in which individuals use language to negotiate power, assert authority, and navigate complex social interactions.

CDA’s focus on the relationship between discourse and social reality also allows for an exploration of how elders adapt to new family structures and societal expectations. It provides a comprehensive view of their experiences and strategies in maintaining family harmony and asserting their rights. In the case of Chinese elders in intergenerational conflicts, CDA helps uncover deeper social and ideological forces, offering insights into the evolving roles and strategies of elders in contemporary society.

This study specifically adopts Fairclough’s [29] dialectical-relational approach to CDA to explore the dynamics of intergenerational family interactions. It focuses on the discursive strategies employed by Chinese elders during family conflicts, aiming to uncover how power relations and social inequalities are reproduced and negotiated through discourse. This approach provides a nuanced understanding of how older adults engage in discourse to assert their rights, navigate family expectations, and redefine their roles within family structures through examining linguistic features, discursive practices, and the broader social contexts shaping these interactions.

Fairclough’s [29] dialectical-relational approach provides a thorough framework for examining how discourse both reflects and shapes power relations, making it particularly relevant for this study on social issues. This approach, drawing from foundational works by CDA researchers such as [34] and further developed by Fairclough [29], emphasizes the nature of discourse with socio-historical and socio-cognitive dimensions. Wodak and Meyer’s discourse-historical approach highlights the importance of historical context in understanding discourse, while van Dijk’s socio-cognitive perspective highlights the mental processes involved in interpreting discourse. Fairclough’s transdisciplinary contributions to CDA enable this framework to explore the complex ways in which discourse reflects and shapes power structures within social settings.

Within Fairclough’s framework, discourse is not merely a neutral channel for communication but a means of constructing reality, deeply influenced by broader socio-economic and political structures as well as specific social practices. Fairclough [35] describes discourse as both ‘the language associated with a particular social field or practice’ and ‘a way of constructing aspects of the world associated with a particular social perspective’ (p. 231). These definitions highlight the dual role of discourse as both a product of social context and a contributor to it. In other words, discourse enables a process of sense-making that is embedded in both cognitive and societal realms, dynamically interacting with other actors and social forces to shape specific narratives and norms.

Above all, this study uses the dialectical-relational approach in CDA to demonstrate how discourse helps build social identities and manage power in different contexts by analyzing intergenerational family dynamics. It connects individual actions with broader social structures, offering valuable insights into how language reflects and shapes power dynamics in various social settings. Specifically, the study also examines the discursive strategies employed by Chinese elders during family conflicts, shedding light on how these strategies are used to assert authority, negotiate expectations, and redefine their own roles within the family structure.

### Data collection and data

The dataset used in this research consists of publicly available recordings excerpted from an Internet platform. As these recordings are openly accessible, no additional permissions or approvals were required from the provider. However, the study strictly adheres to ethical guidelines, ensuring that data usage respects user privacy and follows relevant legal and academic standards.

The current study used a dataset from transcripts of a live television mediation program, offering a valuable source for studying real-life conflicts. This program is notable as the first of its kind in China with legal authority, focusing on resolving conflicts among real people. *The Third Mediation Room* is known for its commitment to authenticity, featuring real cases with actual participants and a genuine mediation process. However, as with other reality-based programs, performativity plays a role in shaping interactions and audience perceptions. Existing research on performativity in reality shows highlights how participants may consciously or unconsciously adjust their behavior in response to the presence of cameras and the structured nature of televised conflict resolution (e.g., [36, 37]. These studies suggest that even in seemingly unscripted settings, participants engage in self-presentation strategies that align with audience expectations and media conventions. Integrating this perspective into the analysis of *The Third Mediation Room* provides a more nuanced understanding of how mediation unfolds in a televised setting, acknowledging both its authenticity and the performative elements inherent in reality-based programming.

Given that all reality shows inherently incorporate elements of performance, our selection process prioritized cases rooted in real-life contexts, particularly those revolving around pressing familial concerns such as property inheritance and elder care—both of which constitute genuine sources of conflict within family dynamics. To enhance the authenticity of our dataset, we meticulously screened the content, deliberately excluding segments that appeared excessively dramatized or overtly staged. Instead, our focus remained on interactions that more faithfully captured the complexities of real-life disputes, as well as the discursive strategies employed by elders and other family members in navigating these tensions. Through the implementation of this selective approach, we ensured that the conflict discourses examined in this study not only reflected actual familial disputes but also adhered to rigorous methodological standards, thereby enhancing the reliability of our analysis.

Within this framework, the present study seeks to explore the strategies Chinese elders employ in reconstructing relationships with family members in intergenerational contexts. The rigorous selection of mediation cases, which serves as a foundational aspect of this research, is governed by four key criteria designed to maximize both the relevance and analytical depth of the study.

First, topic relevance is prioritized, focusing on cases directly related to family conflicts involving older adults, such as inheritance disputes, elder care issues, and late-life remarriage. The selection of cases centered around these specific themes allows the study to closely examine the unique challenges and dynamics that emerge in family relationships involving elders. Second, process completeness is ensured by selecting videos that document the entire mediation process—from the initial presentation of conflict to its resolution. This comprehensive documentation allows for an in-depth analysis of how mediators and family members interact over the course of resolving conflicts. Third, discourse richness is considered, favoring cases with diverse discourse styles and notable emotional changes during mediation. Such variety in discourse offers valuable insights into the ways elders and mediators adjust their communication styles to address and resolve family issues. Finally, representativeness and diversity are achieved by including cases from various regions, cultural backgrounds, and family structures. This approach enhances the study’s applicability, ensuring that findings are relevant across different family dynamics and cultural contexts.

Adherence to these criteria ensures a precise alignment with the study’s central research theme, allowing for a comprehensive and nuanced examination of mediation strategies within familial contexts. This structured approach enables an in-depth analysis of how elderly family members navigate conflicts with other relatives under the facilitation of mediators while also evaluating the effectiveness of their strategic interventions in fostering familial cohesion and sustaining intergenerational harmony.

Following established criteria, the researchers compiled a dataset of 12 video recordings focused on family conflict mediation involving older adults. These recordings were sourced from internet platforms between 2021 and 2022, with the original content in Chinese being publicly available. To ensure analytical accuracy and a faithful presentation of findings, a direct translation approach was used. This method closely adheres to the source text, minimizing potential distortions in meaning. Additionally, the accuracy of the translations was validated through a back-translation procedure conducted by professional translators, further enhancing the reliability of the data.

The collected video recordings span a total duration of 515 min, encompassing approximately 90,000 words. This dataset is sufficiently extensive to encompass a diverse range of conflict types and mediation strategies, while remaining of a manageable scale to facilitate in-depth qualitative analysis.

Throughout the transcription process, a systematic labeling scheme was employed to ensure clarity and consistency: mediators’ statements were uniformly designated with the identifier M, elders’ contributions were marked as E, and disputants’ inputs were labeled as D. To safeguard the confidentiality of all participants, personal names and geographic locations were anonymized across all transcripts.

For data analysis, NVivo14 Pro [38] software was utilized, providing a structured platform for the development of coding schemes and the assessment of inter-rater reliability [39]. Prior to full-scale analysis, two analysts engaged in a comprehensive discussion of the discursive strategies delineated by Li [33], subsequently applying these theoretical constructs to an initial dataset in a pilot analysis, which served as the foundation for establishing the study’s analytical framework.

Building upon this preliminary analysis, the Kappa coefficient test was conducted to quantify inter-rater reliability, yielding a value of 0.942—an indicator of a high degree of agreement [40]. To address minor discrepancies in interpretation, three analysts engaged in iterative discussions to refine and standardize the coding framework. Once a consensus was reached, the first author proceeded with the systematic coding of the remaining dataset in accordance with these rigorously defined criteria.

The finalized coding criteria comprised a meticulously structured set of guidelines collaboratively developed to enhance both consistency and analytical precision. This framework incorporated explicitly defined parameters for each discursive strategy, alongside clearly delineated inclusion and exclusion conditions for coding decisions, thereby ensuring methodological rigor and reliability throughout the analytical process.

### Analytical procedure

**Fig 1:**
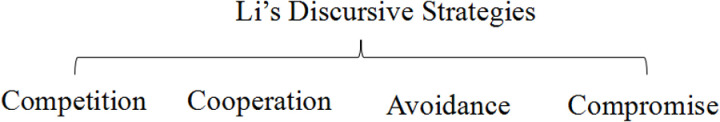
Coding structure.

The analysis of this study employs Li’s [33] discursive strategies. Examples and counterexamples were provided to reinforce the criteria, reducing the chance of misinterpretation. Comprehensive documentation served as an essential reference, ensuring that all analysts applied the coding consistently throughout the analysis. The specific coding structure is illustrated in Fig 1. Additionally, the exploration of discursive strategies and the analysis of power dynamics between elderly individuals and their family members are conducted also using a coding scheme based on [33]. This framework allows for a systematic identification and categorization of various linguistic features to examine the negotiation of power in family interactions. The analysis focuses on how power was asserted, resisted, or redefined through language use, considering both overt and subtle expressions of authority. This approach enabled a comprehensive understanding of the power relations that shape family communication, drawing on the broader social and cultural context of aging and familial roles.

The analytical procedure is based on Fairclough’s CDA framework, which provides a systematic approach to examining the relationship between language, power, and ideology. Fairclough’s model consists of three interrelated dimensions: textual analysis (identification), which focuses on linguistic features; discursive practice (interpretation), which examines the production, distribution, and consumption of discourse; and social practice (explanation), which considers the broader sociocultural context in which discourse is embedded. This framework is particularly useful for exploring the discursive strategies employed by elderly individuals and their family members, as it enables an in-depth analysis of how power is maintained, resisted, or reconfigured through language use. Applying Fairclough’s CDA reveals not only linguistic patterns but also the ideological assumptions underlying family interactions, offering insights into the ways social norms and generational hierarchies shape communicative practices. Detailed procedures can be seen in Table 1.

**Table 1:** Procedure for data analysis.

No.	Phase	Description	Analysis
1	Identification	identifying discursive strategies employed by Chinese elders during intergenerational conflicts in the mediation program.	focuses on spotting how these strategies, such as Competition, Cooperation, Avoidance, Compromise, are used by elders to navigate conflicts. This phase establishes a clear framework for understanding the dynamics of communication in these situations.
2	Interpretation	analyzing how these identified strategies contribute to resolving conflicts and maintaining family harmony.	explains how each strategy reflects the elders’ efforts to assert their authority while also considering the needs of younger generations. This phase highlights the complexities of power dynamics and the interplay between authority and familial relationships.
3	Explanation	clarifying the impact of these strategies on the overall outcomes of intergenerational conflicts in Chinese families.	demonstrates how these strategies influence conflict resolution and promote a sense of respect and understanding among family members. This phase links the findings to broader social implications, emphasizing the role of discourse in shaping familial relationships and cultural values.

## Findings

### Overall analysis of conflict strategies

Through an analysis of the entire dataset, the researchers identified and summarized the distribution of four strategies employed by elders in family interpersonal conflicts. As shown in Table 2, competition and cooperation strategies were the most frequently observed, with respective frequencies of 33.76% and 33.55%. This suggests that elders not only aim to assert their own desires in interpersonal conflicts but also demonstrate a willingness to resolve disputes with the involvement of third-party mediation. Additionally, the avoidance strategy, which accounted for 18.61% of the observed instances, points to a proactive stance taken by elders, who may refrain from unconditionally cooperating with mediators’ inquiries during conflict situations. The compromise strategy, being the least commonly used and comprising only 12.12% of the total, may reflect the fact that compromise typically occurs toward the conclusion of mediation sessions when other strategies have been exhausted. Furthermore, elders may prioritize asserting their rights, which could explain their reluctance to compromise, as they may be unwilling to concede in conflict situations [20].

**Table 2:** Distribution of the use of four strategies.

	Competition Strategy	Cooperation Strategy	Avoidance Strategy	Compromise Strategy
Frequency (Percentage)	165 (33.76%)	155 (33.55%)	86 (18.61%)	56 (12.12%)

### Critical discourse analysis and conflict strategies

#### Competition.

The most frequently used strategy by Chinese elders in family conflicts is the competitive strategy. When individuals feel threatened or attacked, they may adopt a competitive strategy to defend their interests and maintain their dignity. In China, moderate competition is viewed as a social skill that helps individuals establish their position in social interactions [41]. Through clearly stating their stance and needs, individuals can drive discussions and resolve conflicts, ultimately achieving agreement. According to the data collected for this study, elders in family conflicts primarily assert their authority and control through assertion, refutation, and a strong tone.

(1) Assertion

调解人 : 这种被管着的感觉、被控制的感觉特别不好受。对吗?老人家。

老年人 : 这不是管!这是夺权!

M: The feeling of being controlled and managed can be quite uncomfortable, right? Mr.E?

E: This isn’t management! This is a power grab!

Example 1 describes a conflict between a father and son over retirement pensions. The mediator attempts to use empathetic statements to help E acknowledge the discomfort caused by excessive management. The terms *managed* and *controlled* imply a restriction on E’ freedom. However, E firmly rejects this interpretation, asserting, *this isn’t management! This is a power grab*. He denies being managed and instead frames the situation as a power seizure, intensifying the issue.

In the Chinese context, the term *power grab* is highly politicized, but E uses it to express his strong dissatisfaction with his son’s involvement in managing his retirement pension. E feels that his financial autonomy is being violated and believes he has the right to independently control how his pension is used, distributed, and managed. To him, the pension represents not just financial security but also a critical expression of his personal autonomy and decision-making rights. Any effort by his son to affect these decisions appears to be a power grab, revealing E’s awareness of property rights and his strong resolve to protect them. The phrase *power grab* presents the situation as a direct challenge to his authority and independence, highlighting the power struggle and his firm opposition to his son's influence.

(2) Refutation

婆婆 : 天翔工作那么忙,你一天到晚在医院,谁管他?

媳妇 : 他那么大人还要人照顾啊。

婆婆 : 他衣服谁洗?贝贝上幼儿园谁接送?你没点数?

Mother-in-law: Tianxiang is so busy with work, and you’re at the hospital all day. Who’s supposed to take care of him?

Daughter-in-law: He’s an adult; he doesn’t need to be taken care of.

Mother-in-law: Who washes his clothes? Who takes Beibei (daughter) to kindergarten? Don’t you keep up with these things?

Example 2 illustrates a conflict between a mother-in-law and daughter-in-law over household chores. The mother-in-law criticized her daughter-in-law for spending too much time at the hospital taking care of her mother, which led to her rebuff. In this conflict, the mother-in-law displays significant aggression by posing three consecutive challenging questions that rebuff the daughter-in-law’s inappropriate remarks, forcing her to reflect on her role and responsibilities within the family. The first question, *who washes his clothes?* emphasizes the daughter-in-law’s lack of action as a wife. The second question, *who takes Beibei to kindergarten,* highlights her neglect as a mother. The third question, *don’t you keep up with these things,* points to the daughter-in-law’s immaturity as a married woman.

In traditional Chinese culture, elders’ involvement and guidance in family affairs, including the arrangement and supervision of household chores, are crucial to their sense of self-worth and maintaining influence within the family [42]. While the mother-in-law’s three questions seem to focus on specific issues of household labor division, they also touch on deeper aspects of the elders’ perception of family order and their role within the family. The mother-in-law, in rejecting her daughter-in-law’s actions, strategically uses her positional advantage to voice dissatisfaction with the existing division of household tasks. This response serves not only as an attempt to reaffirm or secure her influence in family decision-making but also as a means of reinforcing household practices that align with her own established norms and expectations. Her choice of language reveals a deliberate effort to assert authority, subtly challenging her daughter-in-law’s role. Moreover, through her questioning, she not only directs attention to specific chores but also conveys deeper concerns regarding family roles and the distribution of responsibilities.

(3) A Strong Tone

媳妇 :她做的菜我们吃不惯,老是放太多盐和油。对身体不好。

婆婆 : 我这样吃了一辈子,好好的。不爱吃就不要吃。

Daughter-in-law: We can’t get used to her cooking; there’s always too much salt and oil. It’s not good for our health.

Mother-in-law: I’ve eaten this way my whole life, and I’m just fine. If you don’t like it, don’t eat it.

Example 3 exemplifies a conflict between a mother-in-law and her daughter-in-law concerning dietary practices, wherein the daughter-in-law articulates her dissatisfaction and concerns regarding the mother-in-law’s cooking methods. She particularly critiques the excessive use of salt and oil, arguing that such dietary choices could have detrimental health implications. In response, the mother-in-law asserts her perspective by invoking personal experience, pointing to her lifelong adherence to these eating habits and her own perception of their non-harmful nature. This retort highlights her resolute defense of her culinary practices and conveys a tone of unwavering firmness. The mother-in-law’s remark, *If you don't like it, don't eat it*, implicitly suggests that, rather than attempting to change her cooking style, the daughter-in-law has the option to abstain from eating the food, thereby reflecting a broader sense of autonomy in her stance. This interaction resonates with De Dreu et al.’s [43] argument that competitive strategies in conflict can simultaneously serve as a channel for emotional expression, wherein older individuals may deploy forceful language to convey dissatisfaction or frustration, underscoring the role of language as both a tool of argumentation and a means of emotional release.

The mother-in-law’s response clearly illustrates her determination to protect her lifestyle when facing opposition, demonstrating her firm refusal to accept dietary restrictions imposed by others. She asserts that her eating habits have remained unchanged throughout her life without negative health effects, emphasizing her life experience and expecting recognition of her personal choices from her daughter-in-law. This expectation reflects a deeper demand for understanding and respect toward the unique lifestyle habits of older generations. The discourse further reveals the power dynamics within their relationship, as the mother-in-law insists on maintaining her dietary preferences despite her daughter-in-law’s health concerns. Her language reinforces her identity and autonomy, asserting her right to be respected and understood. A closer analysis of this interaction uncovers the broader social and ideological influences at work, illustrating how language functions as a means of negotiating authority and preserving personal identity within family dynamics.

#### Cooperation.

In interactions, cooperative strategies necessitate that both parties actively engage in collaborative problem-solving by exchanging information and considering diverse perspectives to identify the most effective resolution [33]. Research findings indicate that elderly individuals predominantly employ cooperative strategies by deliberately lowering their own status, transparently disclosing relevant information, and readily acknowledging their mistakes. Through the strategic deployment of such linguistic techniques, elders not only facilitate the resolution of disputes but also subtly assert a degree of control within their exchanges with mediators. In other words, this approach highlights how cooperative language serves as a mechanism through which elderly individuals sustain their agency and exert influence over the conflict resolution process.

(1) Lowering One’s Stance

调解人 :您觉得现在身体不好了,是吗?

老年人 : 现在是路都走不得哦。走到十步,这个腿就痛,那个跟抽筋一样的,拉痛。

M: Do you feel like your health is declining?

E: I can barely walk now. Just ten steps, and my leg hurts, like a cramp, causing severe pain.

In this exchange, the mediator seeks to mitigate the tension in the father-son conflict by strategically introducing a new topic, aiming to cultivate a more harmonious conversational atmosphere. In response, E shows the gravity of his health condition through the statement, *I can barely walk now*, which simultaneously functions as an affirmative reply to the host’s inquiry and a poignant illustration of his current physical predicament. He further elaborates on the discomfort he experiences while walking, vividly likening the pain in his leg to a cramp—a comparison that enhances the immediacy and perceptibility of his suffering, thereby enabling both the mediator and other listeners to grasp his distress with greater clarity and empathy.

Through his deliberate portrayal as an aged and physically debilitated figure, E strategically lowers his status in the discourse, eliciting sympathy while simultaneously strengthening his bargaining position in the familial dispute mediation. This exchange takes place within the broader context of a conflict over pension-related matters, indicating that his remarks serve not only as a reflection of his condition but also as a subtle means of safeguarding his rights, with the implicit goal of persuading his son to loosen financial control. A closer analysis of this interaction reveals the intricate ways in which E manipulates language to influence the mediation process. His detailed depiction of physical suffering functions both as a direct response to the mediator’s inquiry and as a rhetorical strategy designed to evoke empathy and emphasize his vulnerability. The persistent focus on his deteriorating health allows him to frame himself as a figure deserving of care and consideration, thereby indirectly reinforcing his claim to autonomy and asserting his influence over financial matters.

Active Information Sharing

调解人 :后来为什么就过到他名下了呢?

老年人 : 怎么过的?那阵说,得了,你户口回来了,我就把这房给你。谁都没跟他们通知,就归他了。就以后呢,你把我养活死了。我也,他们谁也,没人跟你争。那都各人分各人的房,都没跟他争过。

M: Why did the house end up under his name?

E: How did it happen? At that time, they said, ‘Fine, your household registration is back; I’ll give you this house.’ No one informed them; it just went to him. And then, I told him, ‘You take care of me until I die. I don’t care; no one else will contest with you.’ Everyone had their own houses; no one ever contested it.

In this dialogue, the mediator initiates the exchange with an inquiry, aiming to uncover essential details that will contribute to a more effective resolution of the conflict. E responds with an active engagement in information sharing, demonstrating a remarkable degree of transparency in his communication. The proactive and comprehensive disclosure of information not only facilitates mutual understanding between both parties but also ensures that any potential stakeholders gain a clearer grasp of the situation, thereby establishing a solid foundation for subsequent negotiations [33]. Furthermore, rather than resorting to aggressive or emotionally charged language, E adopts an objective and measured tone, meticulously recounting events and articulating his considerations with precision. This composed and rational communicative strategy serves to mitigate the risk of conflict escalation, fostering an environment conducive to constructive dialogue and cooperative problem-solving.

E’s verbal motivation encompasses multiple interwoven objectives, including the safeguarding of his property rights, the rationalization of his arrangements for elderly care, and the preemptive mitigation of potential familial disputes concerning inheritance, all of which collectively serve to ensure a peaceful retirement free from unwarranted interference. His communicative approach further reflects an implicit expectation that family members will acknowledge and uphold his prior decisions, thereby circumventing conflicts over property and reinforcing a broader demand for the elderly’s right to live without the psychological burden of inheritance-related disputes. A closer examination of his discourse reveals the extent to which E strategically employs language to assert his property rights while simultaneously shielding himself from prospective conflict. Through a composed and logically structured narration of events, he carefully frames his perspective in a manner that underscores the legitimacy of his claims and his insistence on maintaining peace in his later years. This discursive strategy not only highlights E’s efforts to preserve his autonomy but also illustrates his conscious attempts to preemptively counteract any potential challenges to his decisions.

(3) Acknowledging One’s Mistakes

调解人 : 老年人都会养生,应该支持你养生,知道吗?但是,生命在于运动,我们心疼你吃到肚子里边的东西。没病吃出病来。你想想你吃了一肚子,是药三分毒。好吧!

老年人 : 我把这个整个房间里的保健品啊,看作是一种灾难。

M: Older people tend to focus on health; you should also be supported on this. But health comes from exercise. We’re concerned about what you eat; you could get sick from eating too much. Think about it; if you eat a belly full of stuff, remember that medicine can be harmful. All right?

E: I consider all the health care products in this room as a disaster.

Within this interaction, the mediator explicitly articulates the potential hazards associated with an overreliance on health supplements, seeking to prompt E’s recognition of the inherent risks and guide him toward a more balanced perspective. In his response, E not only demonstrates receptiveness to the mediator’s viewpoint but also employs the term *disaster* to acknowledge the severity of his misjudgment, thereby openly admitting his mistake. This act of self-reflection and concession signifies a cooperative communicative stance, one that entails attentively considering alternative perspectives, accepting constructive criticism, and making corresponding behavioral adjustments. Moreover, his deliberate choice of the metaphor *disaster* serves to intensify the rhetorical impact of his statement, effectively conveying the seriousness of the issue while simultaneously assuring the mediator of his comprehension and alignment with the cautionary stance against excessive supplement use.

E’s acknowledgment signifies a deeper realization that, while the pursuit of health is an inherent right of the elderly, an excessive dependence on health supplements may inadvertently compromise their well-being, ultimately contradicting his original intent of securing longevity and vitality. His openness to listening and his willingness to concede personal misjudgments foster an opportunity for enhanced familial harmony, particularly in his relationship with his son. A closer analysis reveals that E’s linguistic choices and verbal responses function as deliberate strategic instruments in navigating power dynamics and exerting influence within the familial sphere. His admission of error, coupled with his acceptance of external critique, underscores not only a conscious effort to preserve familial cohesion but also an implicit commitment to self-improvement and behavioral adjustment. Furthermore, his intentional use of the term *disaster* serves to magnify the perceived gravity of the situation, reinforcing the profound implications of his prior actions and demonstrating the extent to which he recognizes their unintended consequences.

#### Avoidance.

In China, where family interdependence is highly valued, avoidance can be a strategic way to prevent conflicts from escalating. This approach is often observed in marital or coworker disputes [44]. In the Chinese cultural context, direct confrontation may be considered impolite or disrespectful, especially within family settings. The avoidance strategy helps individuals preserve their image and dignity, reducing the risk of negative social evaluation [45]. Moreover, avoidance strategies are closely related to the concept of filial piety, which emphasizes respect, obedience, and harmony within the family. Through the avoidance of direct confrontation, individuals effectively demonstrate a nuanced deference to family elders, thus preserving familial harmony and fulfilling their filial obligations. This strategy allows younger family members to express disagreement in an indirect manner, while still adhering to the social expectation of showing respect to authority figures. In this context, personal feelings are balanced with cultural norms rooted in filial piety, highlighting the delicate negotiation between individual emotions and the societal imperative of hierarchical respect.

For Chinese elders in family conflicts, using avoidance reflects their lower power within the family but also shows their wisdom in protecting their interests and keeping family harmony. In collectivist cultures, like China’s, keeping relationships intact is often more important than resolving conflicts directly [46]. Elders use avoidance strategies like vague language, refusing to answer, or humor-based responses to gain understanding and support without increasing tensions. While Western researchers may see avoidance as an ineffective way to solve conflicts, as it avoids direct confrontation [47], it can be effective in collectivist cultures, where it helps balance personal boundaries with family expectations [48]. Seniors' choice of avoidance allows them to navigate family power dynamics and assert their agency, reflecting the study's CDA framework as it demonstrates their ability to shape conflict resolution while preserving family harmony.

(1) Vague Language

调解人 :老先生,您找邻居那个亲戚朋友借钱是为什么呢?

老年人 : 就是人生在世嘛,总有紧张的时候。借也没借几多钱。

M: Sir, why did you borrow money from a neighbor or a relative?

E: Well, in life, there are always times when you are hard up. I didn’t borrow much.

Within this conversation, when M inquiries about the reason behind E’s decision to borrow money, E deliberately employs vague language in his response. The phrase *in life* constitutes a broad and indeterminate temporal reference, encompassing all phases of an individual’s existence without delineating a specific borrowing period or providing a detailed contextual background. Similarly, the statement *there are always times* when you are hard up circumvents a direct explanation by replacing a precise rationale or explicit purpose for borrowing with a generalized notion of financial hardship encountered over the course of life. Additionally, the use of the vague quantifier *not much* further contributes to the strategic obfuscation of details, as it refrains from specifying the actual magnitude of the debt and withholds concrete numerical values that could invite further scrutiny or discussion. Through this calculated use of linguistic imprecision, E effectively minimizes the level of transparency in his response while subtly steering the conversation away from a direct confrontation regarding his financial choices.

The deliberate use of vague expressions serves as a communicative strategy that effectively minimizes the likelihood of provoking aggressive responses from others, thereby reducing the potential for conflict escalation. This linguistic approach reflects an effort by elderly individuals to safeguard their personal interests while simultaneously maintaining a delicate balance in interpersonal interactions. For instance, E’s employment of ambiguous language not only circumvents the necessity of providing a direct justification for borrowing money but also subtly reframes his financial predicament within the broader and more universally relatable context of life’s inherent challenges. Such an approach diminishes the probability that others will probe further or subject him to criticism, thereby allowing him to navigate the conversation with greater discretion. E’s strategic use of language, coupled with his intentional avoidance of explicit clarification, exemplifies his underlying intent to exert control over the discourse while preserving his social standing. Through the deployment of vague expressions, elderly individuals subtly assert their agency within conversations, maintaining a level of conversational dominance while simultaneously exhibiting a cautious and defensive posture in navigating power dynamics.

(2) Refusal to Answer

调解人 : 我可以自己做主。你刚才说了,是因为他们选了您做这个形象代言人,对吧?你有没有问他们为什么选您做形象代言人?

老年人 : 那我就不知道了。

M: I can make my own decisions. You mentioned earlier that they chose you to be the brand ambassador, right? Did you ask them why they selected you for this role?

E: That, I don’t know.

In this exchange, E’s response to M’s inquiry is marked by a succinct and unambiguous declaration, *That, I don’t know*. This direct refusal to provide an answer indicates not only his unwillingness but possibly his inability to engage with the question of why he was selected as the brand ambassador, thereby creating an immediate boundary around the topic. Such a response effectively curtails any further discussion or potential conflict, as it serves as a clear signal of his disinterest in probing deeper into the issue at hand. While E’s refusal remains firm, it is delivered in a manner that is notably calm and free from overtly confrontational or aggressive language. This measured response helps sustain a peaceful conversational atmosphere, subtly reinforcing his intention to avoid conflict, while maintaining a degree of politeness in the exchange. The strategic mildness of his refusal thus allows him to navigate the conversation with finesse, preventing any escalation of tension while preserving the social harmony of the interaction.

E’s refusal to engage successfully prevents further inquiry into the details of the selection process and his interaction with it. This not only avoids possible follow-up questions or controversies that could arise from disclosing too much information but also offers a degree of privacy and protection for information he may prefer not to disclose. we can interpret E’s avoidance of the question as a strategic maneuver to navigate power dynamics within the family. Refraining from engaging with the question, E may be attempting to assert control over the conversation and preserve a sense of independence, while simultaneously avoiding potential conflict or criticism. His response reflects a broader cultural understanding of the importance of harmony in family relations, a value deeply embedded in many societies, particularly within the context of East Asian traditions. It suggests that discourse functions not merely as a communication tool but as a strategic resource for managing interpersonal relationships and exerting influence in family dynamics. Through the use of specific discursive strategies, elderly individuals navigate the complexities of their interactions with the goal of maintaining familial balance and respect. This suggests that, while they may not always hold overt power, their role within the family is significant in shaping family dynamics. However, it also indicates that elderly individuals often occupy a relatively passive position in power structures, relying on subtle forms of influence rather than direct authority. Such strategies highlight the delicate negotiation of power, respect, and filial piety in intergenerational relationships.

(3) Humor-Based Responses

调解人 :您不觉得他们是担心您,怕您受骗?您不这样想吗?

老年人 : 这个鲁迅也讲过,有缺点战士,他毕竟是战士。五光十彩的苍蝇毕竟是苍蝇。

M: Don’t you think they’re worried about you, afraid you’ll be deceived? Don’t you see it that way?

E: As Lu Xun said, a flawed warrior is still a warrior. A flashy fly is still a fly.

In this conversation, when M probes E about whether he believes others might be concerned about his potential deception, E responds by invoking a metaphorical allusion to Lu Xun, the esteemed Chinese writer and intellectual. The phrase *a flawed warrior is still a warrior* encapsulates the notion that, despite imperfections or shortcomings, an individual’s intrinsic worth or strength remains unaltered, underscoring the enduring value of one's character or position. In contrast, E juxtaposes this with the metaphor *a flashy fly is still a fly*, which conveys the idea that external appearances can be illusory and ultimately fail to reflect the true essence of an individual. This contrast between the two metaphors serves as E’s subtle critique of superficial judgments, suggesting that, regardless of outward impressions or superficial allure, one’s core nature or intrinsic qualities remain unchanged. Through this layered use of metaphor, E not only engages in a form of intellectual discourse but also cleverly navigates the conversation, indicating a deeper understanding of the complexities of perception and reality.

E’s use of metaphor in this context serves a multifaceted purpose, intricately reflecting the host's inquiry while simultaneously offering a perspective that deliberately circumvents a direct answer to the underlying concern. Through the use of metaphors, E frames his response in a manner that emphasizes resilience and a firm sense of self-worth, subtly suggesting that, despite any flaws or potential deception, he perceives himself as akin to a warrior—undiminished in essence. This approach not only enables him to preserve his dignity and authority but also effectively deflects further questioning or probing into his personal beliefs regarding the motives of others. Moreover, by invoking the wisdom of a revered cultural figure like Lu Xun, E lends weight to his argument, seamlessly connecting his position to traditional Chinese values and intellectual insight, thereby elevating the rhetorical strength of his point. In this way, his rhetorical strategy allows him to communicate his perspective indirectly while maintaining the harmony of the conversation.

Humorous responses, as noted by [49], often provide individuals with a way to sidestep contentious or problematic issues, thereby creating more favorable conditions for eventual resolution. Through humor, E skillfully avoids offering a direct answer that might provoke interpersonal tension, instead conveying his self-awareness and personal stance in a manner that is lighthearted yet meaningful. Furthermore, this humorous approach subtly reminds both the mediator and his son of the fundamental principle that elders have the right to make their own decisions, emphasizing that age should not be seen as a limitation to one's ability to discern right from wrong. The use of humor, particularly in the form of metaphor, thus serves as a sophisticated rhetorical device that not only enables the less powerful party to attract greater attention and understanding within the conversation but also strategically diverts the focus from potential conflict. In this way, E’s metaphorical response creates a protective barrier, shielding him from further confrontation while enabling him to retain control over the discourse, ultimately emphasizing his agency in the familial exchange. Additionally, through metaphors rich in cultural and intellectual depth, elders subtly assert their wisdom, cultural literacy, and moral discernment, recalibrating the power dynamics within the family and fostering an environment where their authority and experience are more readily acknowledged. Ultimately, E’s indirect and humorous response highlights the cultural perception of elders as not only deserving of respect but also as individuals capable of navigating complex moral and social issues, thereby reinforcing their position within the family hierarchy.

#### Compromise.

Compromise strategies typically necessitate that all parties involved make certain concessions, thereby ensuring that each individual feels satisfied with the final resolution. The reciprocity inherent in these strategies plays an important role in facilitating effective conflict resolution. For elderly individuals, employing compromise strategies reflects a pragmatic approach to managing familial conflicts, particularly within the context of the prevailing family power structure. These strategies are instrumental in constructing and maintaining specific familial relationships through the discursive practice of compromise. As revealed by the data gathered in this study, Chinese elders commonly resort to compromise strategies that encompass conditional concessions, pseudo-threats, and the offering of compromise solutions. These strategies are intricately linked to the concept of filial piety, as they embody a delicate balance between respecting the authority of elders and maintaining harmony within the family. Filial piety, which emphasizes obedience, respect, and care for one’s parents and elders, often requires younger family members to adjust their behavior to avoid direct confrontation or overt challenges to the hierarchical family structure. In this regard, compromise strategies enable both elders and younger family members to navigate disagreements while circumventing the direct violation of traditional familial norms. This approach facilitates the preservation of family cohesion, subtly renegotiating the power dynamics that underlie familial interactions. The power relations within the context of compromise strategies are marked by a dynamic interplay; although elders may outwardly appear to maintain authority, the act of compromise signals a shift toward more reciprocal, negotiated forms of influence. Such a shift suggests that both parties, in fact, exercise agency: elders use compromise as a means to sustain their influence without rigidly imposing their authority, while younger members engage in compromise as a way of asserting their autonomy while still adhering to their filial obligations.

(1) Conditional Concessions

调解人 : 哪怕就是错的,他们也得让你犯这个错。是吗?

老年人 : 即使犯错,你也要允许我一个改的过程。

M: Even if it’s wrong, they should let you make that mistake, right?

E: Even if I make a mistake, you should allow me the opportunity to correct it.

In this conversation, M, through the use of the tag question *right?*, subtly invites E to engage in a discussion that revolves around the concept of conditional concessions, seeking to establish a point of common ground between them. E’s response, *even if I make a mistake, you should allow me the opportunity to correct it*, begins with an acknowledgment of the premise that human beings are inherently fallible, and mistakes are an unavoidable part of life. However, E goes beyond merely accepting the possibility of error by articulating an additional condition: that the opportunity to rectify mistakes is essential, suggesting that this act of correction is not only a right but a necessary component of resolving any misstep. In this way, E’s response not only aligns with the mediator’s implied invitation to negotiate through conditional concessions but also expands the scope of the discussion by introducing a further requirement—a call for allowance and understanding in the face of error. The use of the first-person pronoun *I* serves to personalize this argument, grounding the assertion in E’s own perspective, thus imbuing his statement with a greater degree of emotional resonance and personal investment. This choice strengthens the persuasive nature of his argument, making it not only a logical appeal but also an emotional one, which can influence the tone of the interaction and potentially shift the mediator’s or M’s stance.

E’s statement can be seen as a reaffirmation of his commitment to respecting the fundamental rights of elderly individuals, which include not only the right to equality but also the inherent need for support from both family and community. In the context of social life, elderly individuals are entitled to the same basic rights as others—rights that encompass the freedom to think independently, make decisions, and bear the consequences of those decisions, including the right to correct mistakes. In this regard, E’s conditional concession can be interpreted as a broader assertion of his autonomy and his insistence on actively participating in social life, thereby resisting any limitations or discrimination based solely on his age. Through this response, E advocates for a more dignified and self-determined existence, wherein elderly individuals are not merely passive recipients of care but are instead empowered agents with the right to make choices and amend their errors.

From the perspective of CDA, this response resonates with the approach of exploring how discourse reflects, constructs, and even challenges prevailing power dynamics, social identities, and hierarchical structures [29]. Emphasizing the need for an opportunity to correct mistakes, E articulates a stance that demands respect, not solely as a reflection of his age, but as a human being deserving of dignity, regardless of his position within the familial or societal hierarchy. This perspective suggests that elders, like all individuals, should have the space to exercise their agency and independence, viewing mistakes as not signs of weakness, but as integral aspects of personal growth and self-determination.

Moreover, E’s response is situated within the cultural framework that shapes familial and societal expectations, which, in turn, influence intergenerational relationships. In championing this perspective, E challenges the conventional hierarchical power structures that have traditionally governed family dynamics, where younger members are expected to show deference and respect toward elders. Through this, E questions the traditional authority that elderly individuals hold within the family unit, an authority often perceived as passive and subordinated to the needs and decisions of younger generations or other societal figures. E’s discourse serves not only as an assertion of his own rights but also as a call for a redefined power balance—one that underscores the agency of elderly individuals. In this way, he shifts the focus from a one-sided, hierarchical relationship to one characterized by balance, reciprocity, and mutual respect, where the elderly are empowered to assert their rights and influence within the family structure.

(2) Pseudo-Threats

In interpersonal conflicts, a pseudo-threat generally refers to a scenario in which one party intentionally fabricates or amplifies a threat that does not genuinely exist or is exaggerated, with the objective of influencing, manipulating, or coercing the other party into making concessions or modifying their behavior. This strategy often relies on intimidation, deception, or psychological manipulation to achieve specific benefits or advantages without overtly exercising the implied power of the threat. However, it is crucial to recognize that family harmony may not be solely preserved through the pseudo-threat itself, but rather through the passive approach adopted by the family member who yields to the elderly, thereby alleviating the tension. This passive deference may serve a vital role in maintaining family unity, as the individual embroiled in the conflict may choose to accommodate the elderly to avert escalation, thus preventing confrontation and safeguarding peace within the family. To more effectively support the argument that the elderly play a pivotal role as conflict resolvers, it would be advantageous to present a more specific example illustrating how their position within the family dynamic contributes to the resolution of such conflicts.

媳妇: 我们两夫妻的事情我们自己知道,你为什么总是干涉我们?

婆婆 : 我管不了了,你们自己的日子,你们自己混下去吧。

Daughter-in-law: We know about our own marriage. Why do you always interfere?

Mother-in-law: I can’t handle it anymore. You live your own lives, and sort it out yourselves.

In this conversation, the daughter-in-law articulates her dissatisfaction with the mother-in-law’s persistent interference in their marital affairs by posing the question, *why do you always interfere?* The tone of her query carries an implicit warning, suggesting that if the situation persists, it could lead to heightened conflicts. This reflects her desire for greater autonomy and control within her own household.

The mother-in-law’s response, *I can’t handle it anymore*, ostensibly conveys a commitment to cease interfering in the couple’s lives going forward. However, this statement functions as a pseudo-threat, as, despite appearing to relinquish her influence, the mother-in-law does not genuinely intend to entirely sever her control over family matters. This remark reveals her underlying dissatisfaction while simultaneously hinting at her continued desire to maintain a degree of authority within the family structure.

When the mother-in-law states, *sort it out yourselves*, she subtly implies that should the couple persist in their current way of living, they are likely to encounter difficulties, disorder, or even failure. This communication of a negative expectation can be interpreted as a veiled threat, signaling the potential consequences of failing to resolve their ongoing conflicts. It suggests that unless they act to rectify their situation, the fallout could be detrimental to their well-being.

In the context of traditional Chinese culture, elders, particularly mothers-in-law, traditionally occupy a higher status and exert significant influence over familial affairs [50]. Even in contemporary society, these traditional values persist, influencing familial interactions. The mother-in-law’s statement reflects an awareness that her continued interference might exacerbate tensions with her daughter-in-law, disrupt family harmony, and ultimately affect her own quality of life. Through the use of pseudo-threats, the mother-in-law conveys her dissatisfaction while attempting to reassert her authority in a manner that deters further conflict without significantly escalating it. This strategy effectively preserves the semblance of control while navigating the delicate balance between maintaining familial influence and avoiding overt confrontation.

The mother-in-law’s statement reflects her acknowledgment of her inability to continue interfering in the lives of her daughter-in-law and son, recognizing that she lacks the legitimate authority to encroach upon the couple’s independent living space and decision-making autonomy. This acknowledgment signals a shift in family power dynamics, where traditional hierarchical authority gives way to more negotiated forms of influence. She also realizes that persistent interference could intensify conflicts with her daughter-in-law, disrupt the family environment, and ultimately diminish her own quality of life. The use of pseudo-threats reveals a strategic attempt to manage these shifting dynamics. It allows her to withdraw from the dispute while preserving a positive image and subtly maintaining influence. Pseudo-threats function both as an emotional appeal and as a negotiation tactic, promoting family harmony without directly challenging the erosion of traditional authority.

(3) Offering Compromise Solutions

调解人 : 您最希望的养老方式,您希望怎么养老?跟谁一起?

老年人 : 哪怕租一间平房呢!我都去!

M: What is your preferred way of living in your retirement years? With whom would you like to live?

E: I’d be content even if it’s just renting a small house! I’d go for it!

In this dialogue, M initiates a conversation with E regarding her future retirement plans, posing an open-ended question designed to prompt E to articulate her vision for an ideal retirement scenario. This question, with its open-ended nature, invites E to express her preferences and aspirations freely, without imposing any predefined constraints. E’s response, which centers around the idea of renting a small house, reflects a clear desire for independent living, emphasizing both autonomy and a preference for a specific type of residential environment. The suggestion of renting a small house indicates her inclination toward a modest, self-sufficient lifestyle, potentially with less reliance on family or others.

Furthermore, the inclusion of the phrase *even if* reveals E’s willingness to make certain concessions in order to fulfill her ideal living arrangement. The phrase suggests a readiness to accept a less-than-ideal housing condition, recognizing that compromises may be necessary to achieve her desired lifestyle in retirement. In this context, the conditional language used highlights E’s flexibility in pursuing her goals, signifying an understanding that perfection may not always be attainable.

The emphatic tone in E’s concluding remark, *I’d go for it!* reinforces her strong determination and resoluteness. This statement highlights her commitment to her vision, illustrating that she is prepared to embrace the proposed compromise if it leads to the realization of her preferred retirement lifestyle. E’s willingness to express her intentions in such a decisive manner demonstrates both confidence and a clear sense of agency in shaping her future, further suggesting her active role in the decision-making process.

E’s response reveals her keen interest in maintaining an independent and autonomous way of life. This response demonstrates a proactive approach to asserting her right to choose a retirement lifestyle that aligns with her personal preferences, free from excessive interference. Considering that her current living situation might not meet her needs for autonomy and comfort, E seeks to take greater control over her retirement by selecting a living arrangement that reflects her aspirations, thereby enhancing her quality of life in her later years.

## Discussion

In the above sections, the authors discuss the characteristics of discursive strategies employed by elderly individuals in familial interpersonal conflicts and how these strategies facilitate power negotiation within the family. The study reveals that the main strategies employed by Chinese elders in intergenerational conflicts are competition, avoidance, cooperation, and compromise. These strategies play a crucial role in protecting elders’ rights in areas such as material wealth, family decisions, personal lifestyle, privacy, and social status. Elders actively negotiate power relations and challenge or reinforce underlying ideologies within family discourse through the employment of these strategies, thereby demonstrating their agency in shaping intergenerational interactions. These findings resonate with Ma [51], who observed that modern Chinese elders vigorously safeguard their rights. In the subsequent section, the authors will shift their focus to the conflict discursive strategies employed by elders, delving deeper into the ideological implications embedded within these strategies. Following this, the following section will explore how Chinese elders apply these discursive strategies to navigate power dynamics in the context of family conflicts, while simultaneously examining the ideological connotations associated with these strategic resources.

This study adopted and expanded upon Li’s [33] four strategies in conflict discourse, illustrating them with abundant examples. Additionally, several sub-strategies for each category were identified within the specific context of Chinese family conflicts. Notably, our analysis reveals how elders employ these strategies to negotiate power relations and maintain agency in family interactions, offering new insights into the pragmatic and ideological dimensions of elder discourse in interpersonal conflicts.

In traditional Chinese society, the concept of *filial piety* was rigorously upheld, granting elders a significant degree of social and familial status [52]. However, following economic reforms and subsequent societal changes, the intergenerational structure underwent a transformation, leading to more egalitarian relationships where elders no longer retained their historically dominant familial position [53]. In this context, Chinese elders, with their extensive life experience, psychological maturity, and nuanced understanding of interpersonal relationships, demonstrate distinctive characteristics in their use of various conflict management strategies within the family structure.

The shift from traditional family dependency to a more independent role has led elderly individuals to employ *competitive strategies* to cope with the challenges posed by their descendants. This study's findings reveal that Chinese elders often resort to conflict strategies such as assertions, rebuttals, and strong tones, indicating a preference for direct confrontation. These strategies suggest that elders are striving to assert their authority and protect their interests through more overt means. This aligns with Cai’s [20] research, which highlights that Chinese elders predominantly use competitive tactics to safeguard their rights. Such strategies reflect a commitment to preserving their dignity, asserting their entitlement to participate in family decision-making, express their views on issues affecting their well-being, and resist attitudes or behaviors that may marginalize their needs.

This pattern of behavior also reinforces traditional rural culture and social norms, wherein elders are typically expected to occupy roles of authority and decision-making. However, as society continues to evolve, so too do the ideologies in rural areas [42]. The competitive strategies adopted by elders in family conflicts also reflect their struggle to adapt to the transformations of modern society. This indicates that while the role of elders remains crucial, their strategies for asserting influence are evolving in response to the changing social landscape.

Cai [20] suggests that the discursive power of elderly individuals in families may be limited, leading them to adopt more subtle or indirect *cooperative strategies* in their conflict discourse to express their needs and viewpoints while avoiding direct confrontation. The use of *cooperative strategies* reflects a changing relationship model focused on mutual respect and understanding among family members. With a growing societal emphasis on family harmony and support between generations, elders are increasingly inclined to resolve conflicts through communication and negotiation to maintain family unity [54]. In this study, Chinese elders use strategies such as lowering their stance, actively exchanging information, and acknowledging their mistakes to protect their rights. These sub-cooperative strategies align with broader social values that prioritize harmonious family relationships and collaboration between generations, indicating a rethinking of the roles of elders as both transmitters of knowledge and experience and as spiritual supports within the family. The use of these strategies reflects their position within the social power structure and their awareness and adaptation to this structure. Through cooperative strategies, elders take an active role in family dynamics, assert their rights, and adapt to evolving family relationships.

Elders may also use *avoidance strategies* to prevent direct conflicts, helping to maintain a surface-level harmony within the family. This approach can stem from a genuine concern for family stability or reflect traditional social norms that discourage openly discussing family disputes while promoting moral values such as respect for elders and love for the young [55]. In this study, the avoidance strategies identified include the use of vague language, refusal to answer, and humorous responses. From an ideological perspective, using vague language reveals elders’ worries about directly expressing themselves, which could challenge their authority. Refusal to answer allows elders to control the flow of information, helping them maintain or enhance their social status, reflecting their understanding of societal expectations and roles. Humor is seen as a way to create social closeness while maintaining distance; it allows the speaker to express their views without directly challenging others. The use of humor by elders shows their strategic thinking about how to express their personal positions while preserving family harmony. These avoidance strategies not only represent elders’ direct reactions to family conflicts but also reflect their acceptance of social expectations and family roles.

The use of *compromise strategies* in family conflicts often shows that elders are willing to give up some of their interests to achieve harmony. This behavior reflects a broader concern for family well-being and a rational assessment of their rights. In the context of societal and family changes, using compromise strategies highlights values that focus on resolving conflicts by balancing interests and promoting peaceful coexistence [56]. In this study, the compromise strategies used by elders include conditional concessions, pseudo threats, and proposing compromise solutions. These sub-strategies reflect the elders’ efforts to find a balance in family conflicts, allowing them to express their needs and positions while maintaining relationships with younger generations. Furthermore, this demonstrates that elders can protect their rights while considering the needs of other family members, showing a deeper understanding of life’s values, an inclusive perspective, and a greater sense of social maturity and family responsibility.

The analysis offers a nuanced understanding of the ideological implications behind the conflict management strategies employed by Chinese elders. These strategies reflect their adaptation to societal changes and underscore the evolving roles of elders within the family. Through competition, cooperation, avoidance, and compromise strategies, elders seek to preserve their traditional authority while also responding to the need for adaptation. They actively recalibrate power dynamics, navigating confrontations to ensure their voices are heard and respected, all while promoting familial unity and tranquility. Through the strategic selection of different approaches, elders facilitate the transmission of cultural heritage, mediate evolving social standards, and reinforce the continued relevance of filial piety in modern times. In this way, they bridge the past and present, integrating respect and familial duty into the fabric of contemporary family life.

Chinese elders navigate their power relations within family conflicts primarily through a careful balancing act of asserting authority and promoting harmony. They do not directly impose their will but rather use discursive strategies that emphasize deference, respect, and the moral obligations inherent in the family structure. Through pseudo-threats, elders can subtly manipulate the behavior of other family members without overtly threatening their authority, thus maintaining control in a way that aligns with the traditional Confucian value of hierarchical family order. Elders also negotiate power by adopting a passive-aggressive approach, where they may indirectly influence decision-making by reminding younger members of their familial duties, positioning themselves as both authority figures and caretakers of family peace. In many instances, their negotiation of power is achieved through a combination of mediation and subtle persuasion, ensuring that their influence is exercised without overt coercion. The strategic use of these discursive tools enables them to maintain a strong yet non-confrontational role in family dynamics, effectively navigating power relations in a way that sustains both their authority and familial harmony.

## Conclusion

This study, using CDA, examines the discursive strategies that Chinese elders use in intergenerational conflicts, focusing on how these strategies shape discourses aimed at protecting their rights. It investigates why these strategies are employed and what ideological messages they convey. While much has been written about intergenerational conflicts, there is limited research on the role of Chinese elders in this context. This study addresses this gap by exploring the conflict discourses elders use and identifying the range of strategies they employ to promote the idea of an AFS.

The analysis shows that Chinese elders use discursive strategies like competition, cooperation, avoidance, and compromise in intergenerational conflicts. These strategies not only reflect social values that support the protection of elderly rights, family equality, and harmony, but also suggest a rethinking of elders’ roles in society. These strategies highlight a proactive approach to protecting their rights and adapting to changing family structures due to social shifts. The choices made by elders emphasize their desire for self-respect, their efforts to defend their legitimate rights, and their commitment to maintaining family harmony in a changing cultural environment. Furthermore, the study identifies 12 sub-strategies within these four main strategies, such as asserting one's position, lowering one’s stance, and using humor. This expands Li’s [33] strategic framework and enriches the use of CDA in specific cultural contexts.

The use of these strategies reflects two key ideas. First, it aligns with social beliefs that emphasize the protection of elderly rights and the importance of family unity. Second, it demonstrates how elders are adjusting their roles and influence within the family as society changes. These strategies show that elders are actively working to secure their rights within the family and social structures, highlighting the complexity of intergenerational relationships in modern China. They reflect the challenge of balancing traditional values with new family dynamics, as elders work to maintain their authority and roles in a changing society.

Ultimately, the conflict strategies employed by elders serve as critical tools for safeguarding their status and fortifying family bonds, while also carrying significant social implications. Through their active involvement in family dynamics, elders not only contribute to the transformation of family structures but also challenge longstanding norms surrounding aging and authority. These strategies ensure that their voices are both heard and respected, which, in turn, cultivates healthier intergenerational relationships and fosters societal awareness of the roles and rights of the elderly in an era of rapid societal change. However, this study is not without its limitations. The findings are predominantly rooted in specific cultural contexts, thereby potentially limiting their applicability to other societies. Moreover, the relatively small sample size and the qualitative nature of the data may not fully capture the diversity of experiences among elders. To overcome these limitations, future research should aim to include more diverse samples and consider cross-cultural comparisons.
